# The role of melatonin deficiency induced by pinealectomy on motor activity and anxiety responses in young adult, middle-aged and old rats

**DOI:** 10.1186/s12993-024-00229-y

**Published:** 2024-02-27

**Authors:** Jana Tchekalarova, Desislava Krushovlieva, Petj Ivanova, Zlatina Nenchovska, Gergana Toteva, Milena Atanasova

**Affiliations:** 1 Institute of Neurobiology, Bulgarian Academy of Sciences (BAS), 1113 Sofia, Bulgaria; 2 Medical University–Pleven, 1 Kliment Ochridski Str., 5800 Pleven, Bulgaria

**Keywords:** Aging, Pinealectomy, Anxiety, HPA axis, Hsps 70 and 90, Rat

## Abstract

**Background:**

Aging affects anxiety levels in rats while the pineal gland, via its hormone melatonin, could modulate their inherited life “clock.” The present study aimed to explore the impact of plasma melatonin deficiency on anxiety responses and the possible involvement of the hypothalamic-pituitary-adrenocortical (HPA) axis and heat shock proteins (Hsp) 70 and 90 in the frontal cortex (FC) and the hippocampus in young adult, middle-aged and elderly rats with pinealectomy.

**Results:**

Melatonin deficiency induced at different life stages did not affect the lifespan of rats. Pinealectomy abolished the circadian rhythm of motor activity, measured for 48 h in the actimeter, in young adult but not in middle-aged rats. Pinealectomy reduced the motor activity of the young adult rats during the dark phase and impaired the diurnal activity variations of old rats. The same generations (3- and 18 month-old rats with pinealectomy) had lower anxiety levels than the matched sham groups, measured in three tests: elevated-plus maze, light–dark test, and novelty-suppressed feeding test. While the activity of the HPA axis remained intact in young adult and middle-aged rats with melatonin deficiency, a high baseline corticosterone level and blunted stress-induced mechanism of its release were detected in the oldest rats. Age-associated reduced Hsp 70 and 90 levels in the FC but not in the hippocampus were detected. Pinealectomy diminished the expression of Hsp 70 in the FC of middle-aged rats compared to the matched sham rats.

**Conclusions:**

Our results suggest that while melatonin hormonal dysfunction impaired the motor activity in the actimeter and emotional behavior in young adult and elderly rats, the underlying pathogenic mechanism in these generations might be different and needs further verification.

## Background

Literature data in experimental rodents demonstrated an age-associated reduction in motor activity accompanied by an enhanced level of anxiety [1–3]. These behavioral changes, measured in the open field (OF) and elevated plus maze (EPM), were detected in different rat strains, including Wistar, Lewis, and Sprague Dawley rats and both in male and female Wistar rats. However, few reports suggest that rat anxiety levels decrease with aging [4–6]. Clinical data indicates that exacerbated emotional state in aging might be closely related to loneliness and affection [7, 8]. The results considering the link between motor activity and anxiety responses are ambiguous. While Torras-Garcia [6] reported that diminished anxiety in rats was accompanied by decreased motor activity, Turner et al. [9] showed that elevated anxiety in old rats did not accompany changes in locomotor activity. Further, Boguszewski and Zagrodzka [1] reported that anxiety–like behavior was not dependent on motor activity in these tests both in young adult and old rats, while Sudakov et al. [3] suggest that there is a close relationship between the two behavioral parameters and that the direction of changes is the same.

The protective role of melatonin on aging is well-known and closely related to its putative antioxidant and anti-inflammatory activity [10] as well as its modulatory effect on neuronal energy metabolism [11]. Aging is associated with a decline in melatonin synthesis and an impaired circadian rhythm of hormonal production, making the organism vulnerable to neurodegenerative diseases, cancer, or metabolic syndrome [12, 13]. Clinical data showed that plasma melatonin levels drop more than twice with aging [14]. Pinealectomy, known to disrupt melatonin production as a hormone, accelerates aging [15, 16]. However, the studies of Pierpaoli et al. [15], suggested that the acceleration or delay of lifespan depended on the age stage when melatonin deficiency was provoked. Recently, we confirmed the hypothesis of Pierpaoli, et al. [15] and demonstrated that pinealectomy of rats aged three months accelerated the aging process via impairment of crucial biochemical, metabolic, and physiological markers. In contrast, a lack of melatonin production produced negligible results in old rats [17]. Further, Pierpaoli, et al. [15] suggested that removing the pineal gland in 14 month-old rats is a crucial period when the gland has already fulfilled its functions and might delay the aging process. However, our findings suggest that the pineal gland is still essential for balancing endogenous antioxidant and pro-oxidant components in middle-aged (14 month-old) rats [18]. Pinealectomy in young adult rats produced impulsive-like behavior with increased motor activity in a novel environment and reduced anxiety [19]. Emotional disturbance (diminished anxiety and depressive-like responses) in 3-month-old rats with melatonin deficiency was accompanied by an impaired feedback mechanism of corticosterone release [19].

The present study aimed to elucidate the impact of the pineal gland during aging on the emotional status of Wistar rats. Melatonin deficiency was induced through the removal of the pineal gland in young adult rats (3 months), middle-aged rats (14 months), and old rats (18 months). Anxiety level was assessed by three different tests: EPM, light–dark test (LDT), and novelty-suppressed feeding test (NSFT). In addition, short-term motor activity was evaluated in a novel environment, while long-term locomotion was registered in the actimeter. Based on previous literature data [4, 20–23] and our results in pinealectomy [21] regarding the crucial role of hypothalamus–pituitary–adrenal (HPA) axis and heat shock protein (Hsp) 70 and Hsp 90 related to the aging process in experimental rats, specific age generations were chosen for testing and clarifying the mechanisms associated with age-related changes in emotional reactivity, and in the particular age-dependent effect of plasma melatonin deficiency on emotional responses.

## Results

### Experiment #1

**Fig. 1:**
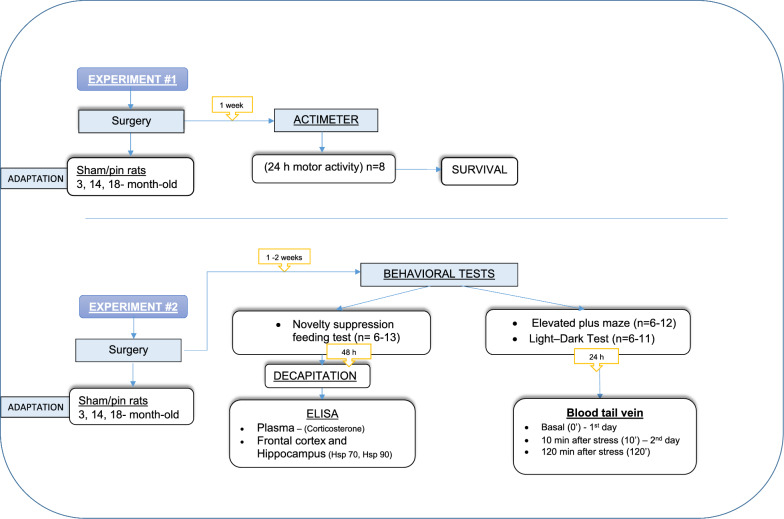
Schematic illustration of the experimental protocol

In Experiment #1, a total of fourty eight animals were used and distributed in six groups (p = 8 per group) (details in Fig. 1 in Methods).

### Pinealectomy reduced the motor activity of the young adult rats during the dark phase and impaired diurnal activity variations of old rats

**Fig. 2:**
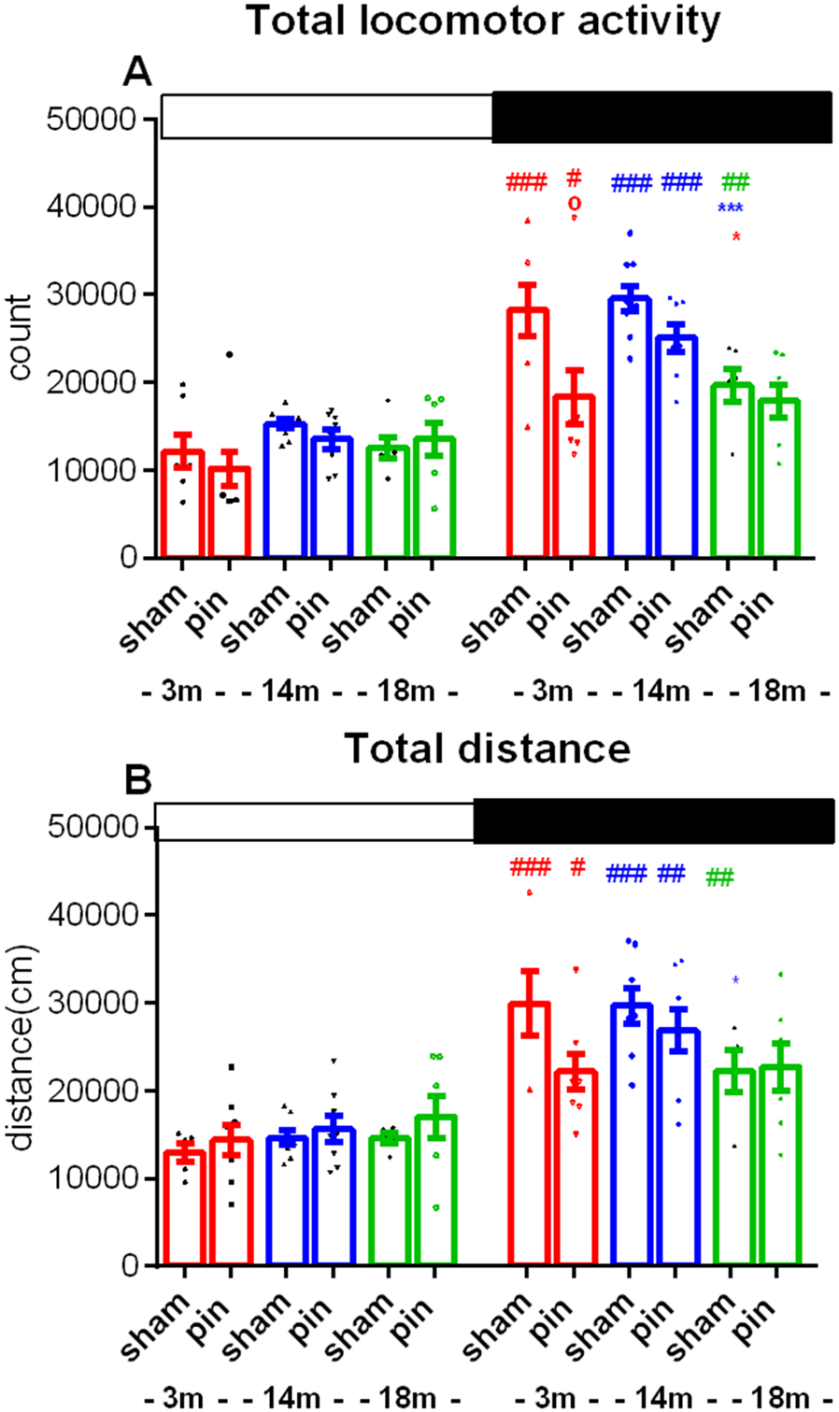
Diurnal rhythm of 24 h registered activity in actimeter (total counts **A** and distance **B**) in the sham- and pin-operated rats aged 3-, 14- and 18 months. The two phases (Light and Dark) are shown with open and black rectangles above the figures. Data are presented as mean ± SEM, n = 8. Three-way ANOVA showed a main Age effect for total locomotor activity [F2,71 = 4.92, p = 0.01], Surgery effect [F2,71 = 3.82, p = 0.026] and Phase effect [F2,71 = 33.42, p < 0.001]. ^#^p < 0.05; ^##^p < 0.01; ^###^p < 0.001 Dark phase compared to the Light phase; ***p = 0.0012, 18 month-old sham rats compared to the matched 14-month-old rats; ^o^p = 0.029, 3 month old pin rats compared to the matched sham rats. **A** *p = 0.012, 18 month old sham rats compared to 14 month-old rats **B**

The first 2 h in the apparatus were eliminated from the calculation for good habituation to a novel environment. Three-way ANOVA indicated that young adult, middle-aged, and old sham groups were generally more active during the dark phase than the light phase (p < 0.05) (Fig. 2A, B). However, pinealectomy abolished diurnal activity in the oldest groups (p > 0.05). The 18-month-old sham group demonstrated decreased locomotor activity (p = 0.035 vs. 3 month-old rats; p = 0.001 vs. 14 month-old rats) and distance (p = 0.012 vs. 14-month-old rats) during the dark phase when the nocturnal rats were active (Fig. 2B). Pinealectomy reduced motor activity in the young adult compared to the matched sham group (p = 0.029) during the dark phase.

**Fig. 3:**
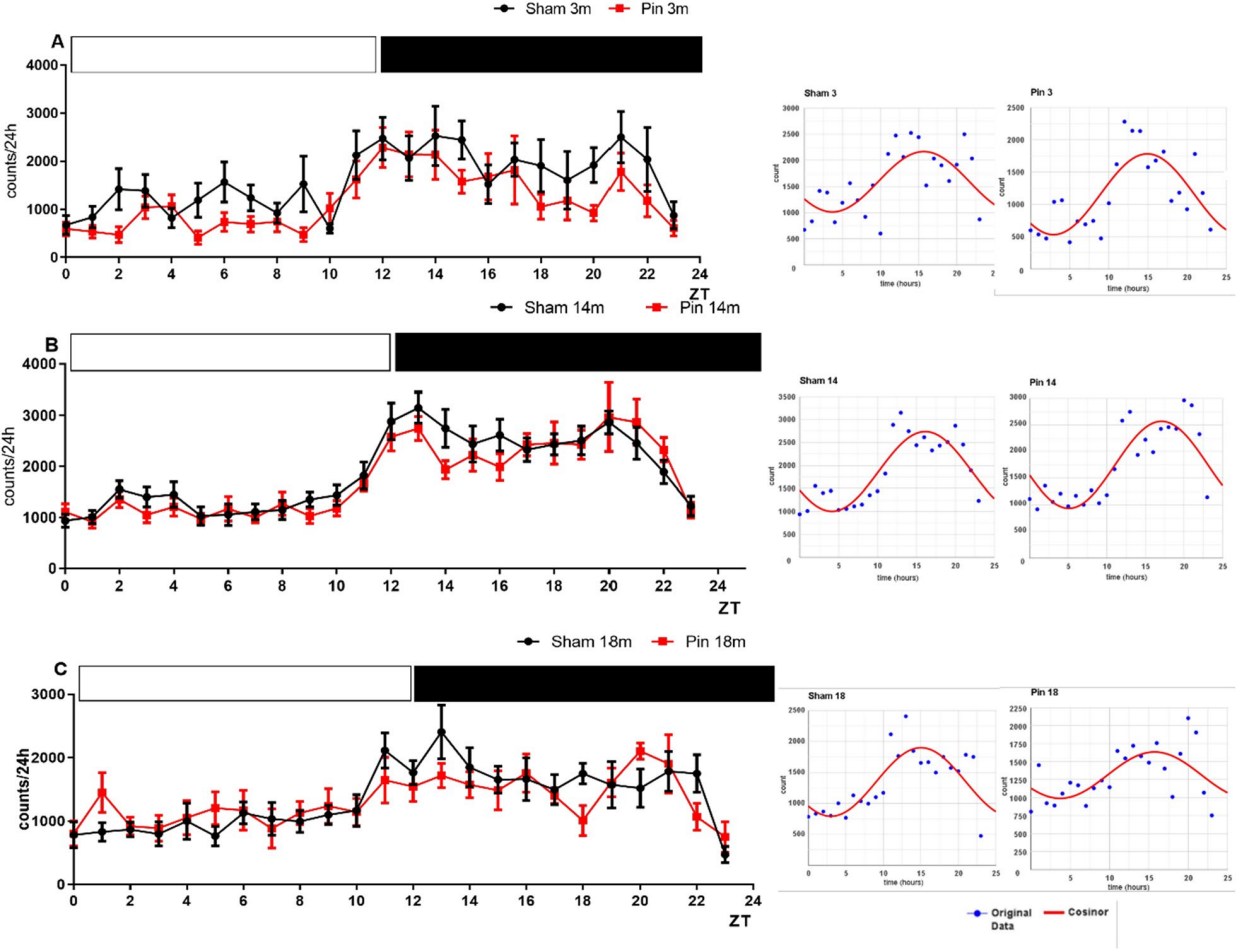
The circadian rhythm of horizontal activity detected in actimeter for 24 h (counts) effect of pinealectomy induced in the sham- and pin-operated rats aged 3- **A**, 14- **B** and 18 months **C**. Data are presented as mean ± SEM, n = 8. Two-way repeated ANOVA demonstrated a main time effect for 3-, 14-, and 18 month-old rats (p < 0.001). Post hoc test showed diurnal variability in motor activity in 3-month-old sham rats (zeigeber (ZT)10 vs. ZT14,ZT21 (p < 0.05) (A), in 14-month-old sham rats (ZT0 vs. ZT 12,13,14,1516,17; ZT1 vs. ZT12,13,16,20,21; ZT3 vs. ZT13; ZT4 vs. ZT13,20; ZT5 vs. ZT12,13,16,19,20; ZT6 vs. ZT12,13,14,15,16,18,19,20,21; ZT7 vs. ZT12,13,20; ZT8 vs. ZT12,13,16,20,21; ZT9 vs. ZT13,20; ZT10 vs. ZT13; ZT23 vs. ZT12,13,14,16,18,20 – p < 0.05), in 14 month-old pin rats (ZT5 vs. ZT19—p < 0.05), and in 18-month-old sham rats (ZT0-ZT4 vs. ZT11; ZT5 vs. ZT11,13; ZT8 vs. ZT13; ZT23 vs. ZT11,13,21–p < 0.05). On the right to the figures are inserted Cosinor data

Two-way repeated ANOVA demonstrated circadian rhythm of activity in the young adult, middle-aged and old sham rats that were not modified by pinealectomy in 3- and 14-month-old rats (Fig. 3A–C). However, the 18-month-old rats with pinealectomy had abolished rhythmic oscillations of motor activity in the actimeter (p > 0.05).

The circadian activity rhythm was also confirmed by the Cosinor analysis in Table 1. The middle-aged sham rats showed significantly higher amplitude values than matched younger rats (p = 0.004).

**Table 1:** Cosinor-based rhythm parameters for the 24-monitored motor activity of Pin and Sham groups, sorted by Age (3 m, 14 m, 18 m); n = 8

	Mesor	Amplitude	Acrophase (hours,decimal)
Sham 3 m	1684.85 ± 301.40	461.48 ± 85.94	15.39 ± 1.03
Pin 3 m	1207.90 ± 232.21	640.72 ± 109.75	14.95 ± 0.63
Sham 14 m	1792.69 ± 47.87	839.50 ± 88.54	16.15 ± 0.51**
Pin 14 m	1666.84 ± 74.21	743.56 ± 78.00	16.46 ± 0.56
Sham 18 m	1345.01 ± 32.50	681.60 ± 80.13	14.24 ± 1.34
Pin 18 m	1311.87 ± 142.22	411.83 ± 55.11	15.93 ± 1.19

Two-way ANOVA indicated a main Age effect for Amplitude [F2,46 = 8.624, p < 0.001] as well as Age x Surgery effect [F4,46 = 2.562, p < 0.05]; p = 0.004 14 month-old sham vs the matched 3-month-old rats

### Melatonin deficiency associated with pineal gland removal does not modify lifespan in young adult, middle-aged and old rats

**Fig. 4:**
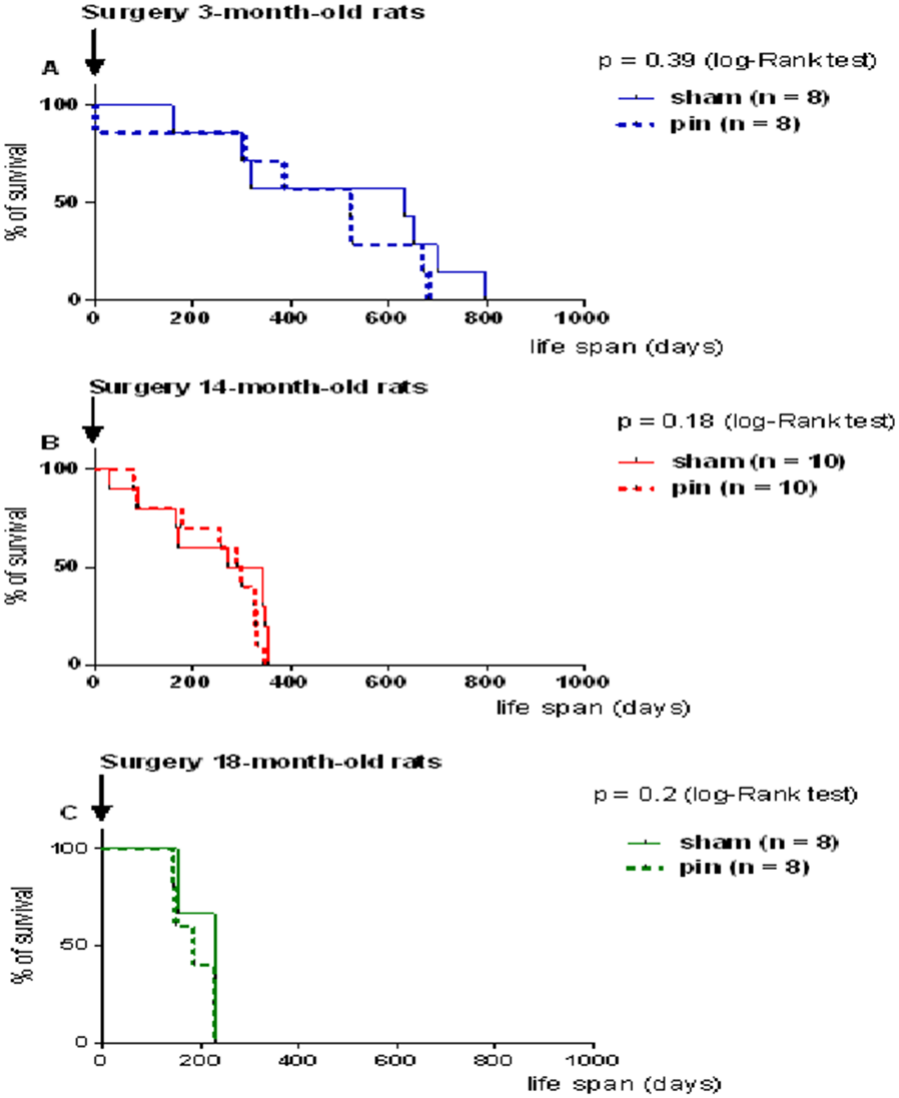
The effect of pinealectomy on lifespan in 3 month-old Wistar male rats **A**, 14-month-old rats **B** and 18 month-old rats **C**. Survival curves (Kaplan–Meier) were not different between sham- and pin-group within each age group, n = 8–10/group, p > 0.05 by log-rank test

The pinealectomy did not affect lifespan and longevity in young adult rats (p = 0.39), middle-aged rats (p = 0.18) and old rats (p = 0.2) (Fig. 4A–C).

### Experiment #2

#### Young adult and old rats with pinealectomy demonstrate impulsive-like behavior and low anxiety level

Various cohorts of animals were employed for each distinct behavioral test, and these cohorts were gathered over a span of 2 years. This meticulous approach to assembling diverse groups of animals allows for a comprehensive understanding of their behaviors under specific conditions. By utilizing different cohorts, we could account for potential variations in factors such as age, genetics, and environmental influences, ensuring a more robust and reliable interpretation of the experimental results.

#### Elevated plus-maze test

**Fig. 5:**
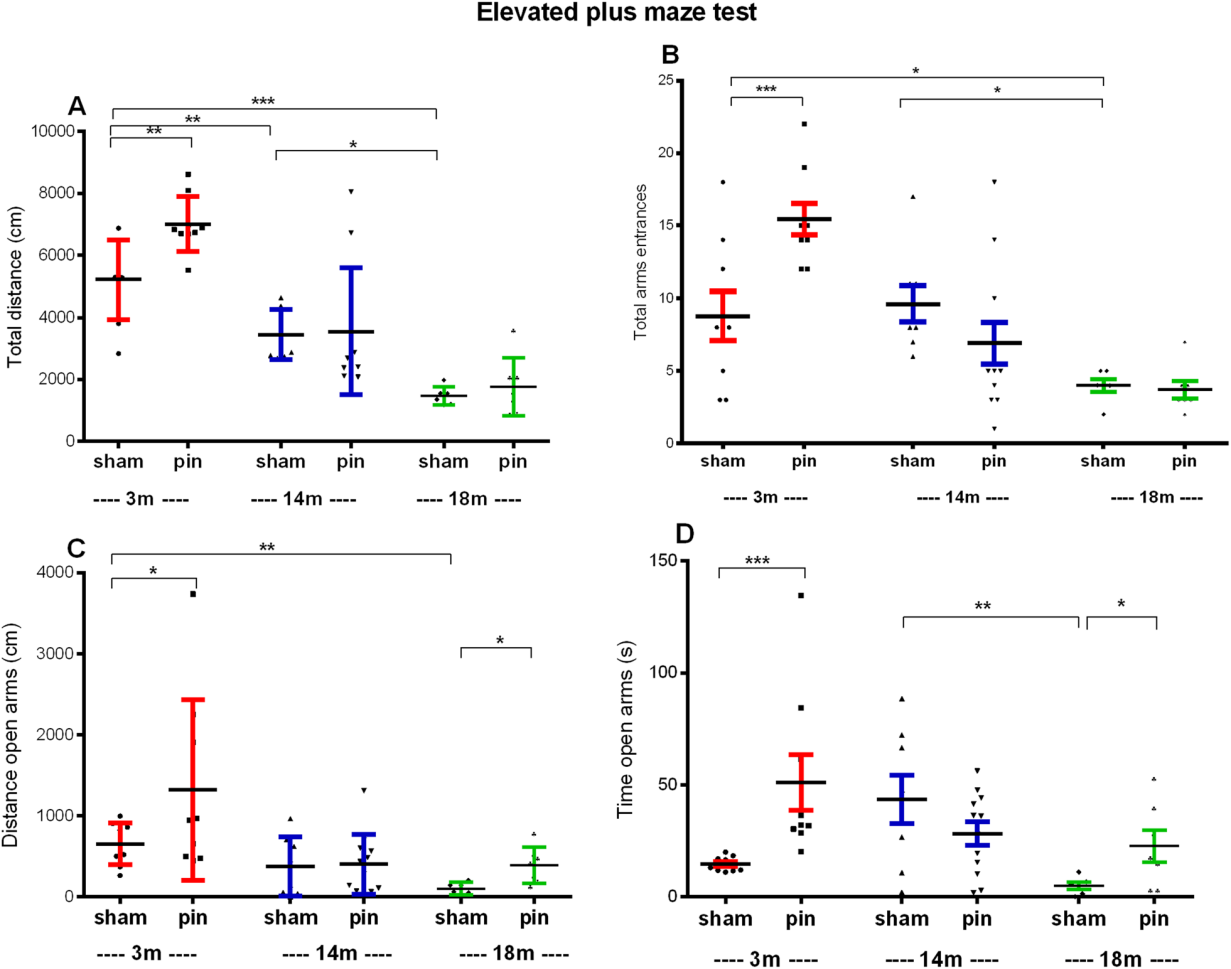
The effect of pinealectomy on total distance **A**, total arms entrances **B**, distance in open arms **C**, time in the open arms **D**, measured in the EPM test, in 3-, 14- and 18 month-old rats measured. Data are presented as mean ± SEM. **A** main Age effect was indicated for total distance [F2,50 = 46.817, p < 0.001], total arms entrances [F2,50 = 17.427, p < 0.001], distance in open arms [F2,50 = 8.725, p < 0.001], time in open arms [F2,50 = 4. 011, p = 0.025]. The main effect of Surgery was significant for distance in open arms [F1,50 = 4.436, p = 0.041], and time in open arms [F1,50 = 4.112, p = 0.047]. Age x Surgery interaction was detected in total arms entrances [F2,50 = 7.280, p = 0.002], and time in open arms [F2.50 = 6.092, p = 0.005]. ***p < 0.001, 18-month-old sham rats compared to 3 month-old sham rats; *p = 0.014, 18 month-old sham rats compared to 14 month-old sham rats; **p = 0.008, 14 month-old sham rats compared to 3 month-old sham rats; **p = 0.005, 3 month-old pin rats compared to matched sham rats **A**; *p = 0.045, 18-month-old sham rats compared to 3 month-old sham rats; *p = 0.028, 14 month-old sham rats compared to 3 month-old sham rats; ***p < 0.001, 3-month-old pin rats compared to matched sham rats **B**; p = 0.01, 18 month-old sham rats compared to 3 month-old sham rats; *p = 0.013, 3 month-old pin rats compared to matched sham ratsp = 0.0126, 18-month-old pin rats compared to matched sham rats **C**; *p = 0.025, 14-month-old sham rats compared to 3 month-old sham rats; **p = 0.009, 18-month-old sham rats compared to 14 month-old sham rats; ^***^p = 0.001, 3 month-old pin rats compared to matched sham rats; *p = 0.045, 18 month-old pin rats compared to matched sham rats **D**

Age-dependent decrease of the motor activity in the novel environment of the elevated plus maze apparatus was detected (p < 0.001, p = 0.008, 3-month-old sham rats compared to 14- and 18-month-old rats, respectively; p = 0.014, 14-month-old rats compared to 18-month-old rats) (Fig. 5A). Total number of entries in the arms was also significantly decreased in the oldest sham group compared to the young adult (p = 0.045) and middle-aged sham group (p = 0.028) (Fig. 5B). The young adult rats with pinealectomy exhibited higher motor activity in the EPM compared to the matched sham rats (total distance: p = 0.005; total arm entries: p < 0.001) (Fig. 5A, B). Concomitant to the age-dependent reduced motor activity, the old sham-treated rats showed decreased distance (p = 0.01 compared to 3-month-old sham rats) and time of entries in the aversive open arms of the EPM (p = 0.009 compared to 14-month-old sham rats) (Fig. 5C, D). Removal of the pineal gland diminished anxiety levels in young adult rats who moved and spent more time in the open arms of the EPM compared to the matched sham group (p = 0.013 and p = 0.001, respectively). Surprisingly, pinealectomy also reduced anxiety in the elderly group compared to their matched sham group, showing an increased distance and time spent in the aversive zone (p = 0.0126; p = 0.045).

#### Light-dark test

**Fig. 6:**
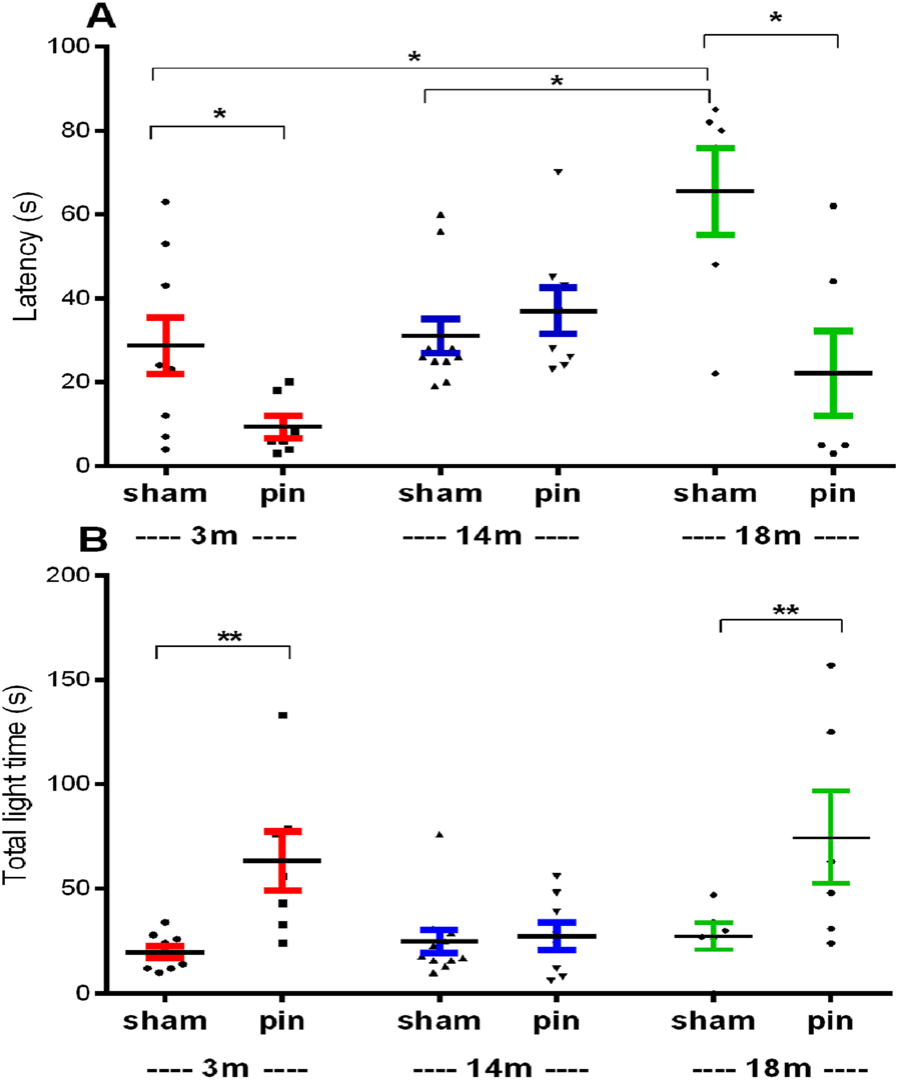
The effect of pinealectomy on latency to cross in the lid compartment **A** and time spent in the lid compartment **B**, measured in the light–dark test, in 3-, 14- and 18 month-old rats. Two-way ANOVA showed a main Age effect [F2,46 = 6.668, p = 0.003]; [F2,46 = 3.242, p = 0.049], Surgery effect [F1,46 = 12.107, p = 0.001], [F1,46 = 14.754, p < 0.001] and Age x Surgery interaction [F2,46 = 6.839, p = 0.003], [F2,46 = 3.436, p = 0.042] for the latency to cross in the lid compartment and time spent in the lid compartment. Data are presented as mean ± SEM. *p < 0.001, 18 month-old sham rats compared to 3-month-old sham rats; *p < 0.001, 18-month-old sham rats compared to 14 month-old sham rats; ^*^p = 0.041, 3 month-old pin rats compared to matched sham rats; ^*^p < 0.001, 18 month-old pin rats compared to matched sham rats **A**; ^**^p = 0.003, 3 month-old pin rats compared to matched sham rats; ^**^p = 0.004, 18 month-old pin rats compared to matched sham rats **B**

The age-associated elevation of anxiety responses was also confirmed in the LDT where the elderly sham-treated rats exhibited longer latency to move to the aversive lid compartment compared to the young adult sham rats (p = 0.017) and middle-aged sham rats (p = 0.015) (Fig. 6A). Diminished anxiety response was also demonstrated as a result of the removal of the pineal gland of 3-month-old and 18-months rats that had lower latency to cross in the aversive compartment (p = 0.032 compared to 3-months old sham rats; p = 0.014 compared to 18-months old sham rats) and spent more time in the light compartment (p = 0.003 and p = 0.004 compared to sham rats, respectively) and (Fig. 6A, B).

#### Novelty suppression feeding test

**Fig. 7:**
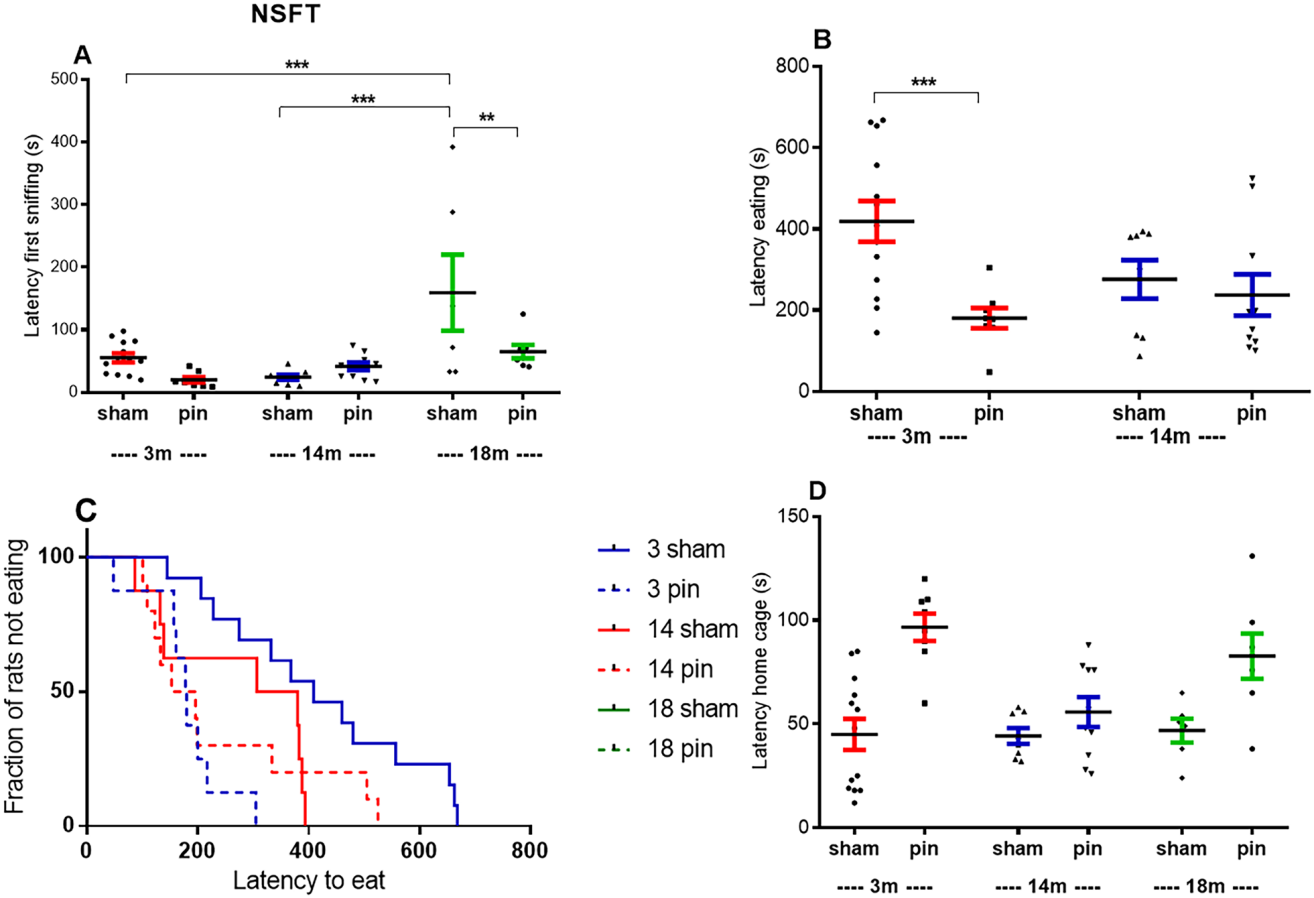
Effect of pinealectomy on latency to start sniffing the food pellets **A**, latency to start eating in the open field **B**, fraction of rats not eating **C**, latency to start eating in the home cage **D**, measured in the novelty suppression feeding test, in 3-, 14- and 18-month-old rats. Two-way ANOVA revealed a main Age effect [F2,56 = 10. 673, p < 0.001]; [F2,56 = 22.025, p < 0.001], Surgery effect [F2,56 = 8.041, p = 0.007], [F2,56 = 5.291, p = 0.026] and Age x Surgery interaction [F2,56 = 4.239, p = 0.02], [F2,56 = 4.410, p = 0.018] for the latency to start sniffing the pellets and the latency to start eating in the open field, respectively. A significant difference was found using the log-rank test for the survival curves (Kaplan-Meier) (p = 0.0048). Data are expressed as mean ± SEM. ***p < 0.001, 18-month-old sham rats compared with 3-month-old sham rats; ***p < 0.001, 18-month-old sham rats compared with14-month-old sham rats; **p = 0.002, 18-month-old pin rats compared with matched sham rats (**A**); ***p < 0.001, 3 month-old pin rats compared with matched sham rats (**C**).

Three parameters in the NSFT assessed the anxiety level: latency to start sniffing the food pellets in the circle of the novel environment and latency to eat the food pellets (Fig. 7A, B). The latency to start eating the pellets in the home cage was also estimated to distract the behavior in the novelty from the place where the rats were well accommodated and were not expected to express anxious reactions [24]. Like in the EPM and the LDT, the 18-month-old rats exhibited increased anxiety response with increased latency to start sniffing compared to the young adult rats (p < 0.001) and the middle-aged rats (p < 0.001) (Fig. 7A). Data of 18-month-old rats describing latency to start eating were not included in the analyses because of the lack of animals starting to eat. Therefore, the Kaplan–Meier cumulative survival curve was used to demonstrate the age and pinealectomy-related fraction of animals that did not eat during the test (Fig. 7C). Melatonin deficiency, related to the removal of the pineal gland, induced a reduced level of anxiety in the young adult rats (p < 0.001, p = 0.0048 compared to their matched sham group, respectively) (Fig. 7B, C) and elderly rats (p = 0.002 compared to their matched sham group) (Fig. 7A).

### Pinealectomy exacerbates the basal levels of plasma corticosterone in old rats

Blood samples were collected from each group at basal conditions (before the stress procedure).

(‘0), 10 min after exposure to forced swimming for 5 min (10’), and 120 min after stress (120’) to assess the activity of the HPA axis and the potency of feedback mechanism responsible for maintaining plasma corticosterone in normal values. Corticosterone levels in plasma were elevated in elderly sham rats compared to young adult rats (p < 0.05) (Table 2). There was no age-dependent changes in the activity of the HPA axis verified by the lack of difference in plasma corticosterone levels at 10’ and 120’ after stress. Removal of the pineal gland induced an additional rise in the plasma corticosterone in the 18-month-old rats (p < 0.05 compared to the matched sham group). Expect for the 18-month pin group, all tested groups showed a significant stress-induced increase in corticosterone (p < 0.05). Two hours later, the hormonal levels were reduced to basal levels, suggesting an intact inhibitory feedback mechanism of the HPA axis, which was unaffected by age and melatonin deficiency.

**Table 2:** Effect of pinealectomy, induced in 3-, 14- and 18 month-old rats, on plasma corticosterone level (ng/ml) in basal condition (0ʹ), 10 min (10ʹ) and 120 min after stress procedure (forced swimming for 5 min)

Time	Corticosterone (ng/ml)		
	0ʹ	10ʹ	120ʹ
Group			
Sham 3 m	9.042 ± 0.126	15.44 ± 1.925*	5.118 ± 0.199^#^
Pin 3 m	8.860 ± 0.223	15.38 ± 1.520*	5.188 ± 0.163^#^
Sham 14 m	9.114 ± 0.348	16.05 ± 0.975*	5.281 ± 0.137^#^
Pin 14 m	8.781 ± 0.263	16.60 ± 1.731*	5.221 ± 0.137^#^
Sham 18 m	9.362 ± 0.239^**+**^	17.24 ± 1.364*	5.331 ± 0.124^#^
Pin 18 m	15.09 ± 1.78^o^	17.38 ± 1.668	5.284 ± 0.128^#^

Data are given as mean ± SEM and analyzed by three-way ANOVA followed by Bonferroni post hoc test (n = 6–8). *p ≤ 0.05 in comparison with 0ʹ within the same group; + p ≤ 0.05 in comparison with the 3 month-old sham rats; ^#^p ≤ 0.05 in comparison with 10ʹ; ^o^p ≤ 0.05 in comparison with the matched sham group at the same age

### Middle-aged rats with pinealectomy have reduced expression of Hsp 70 in the frontal cortex

**Fig. 8:**
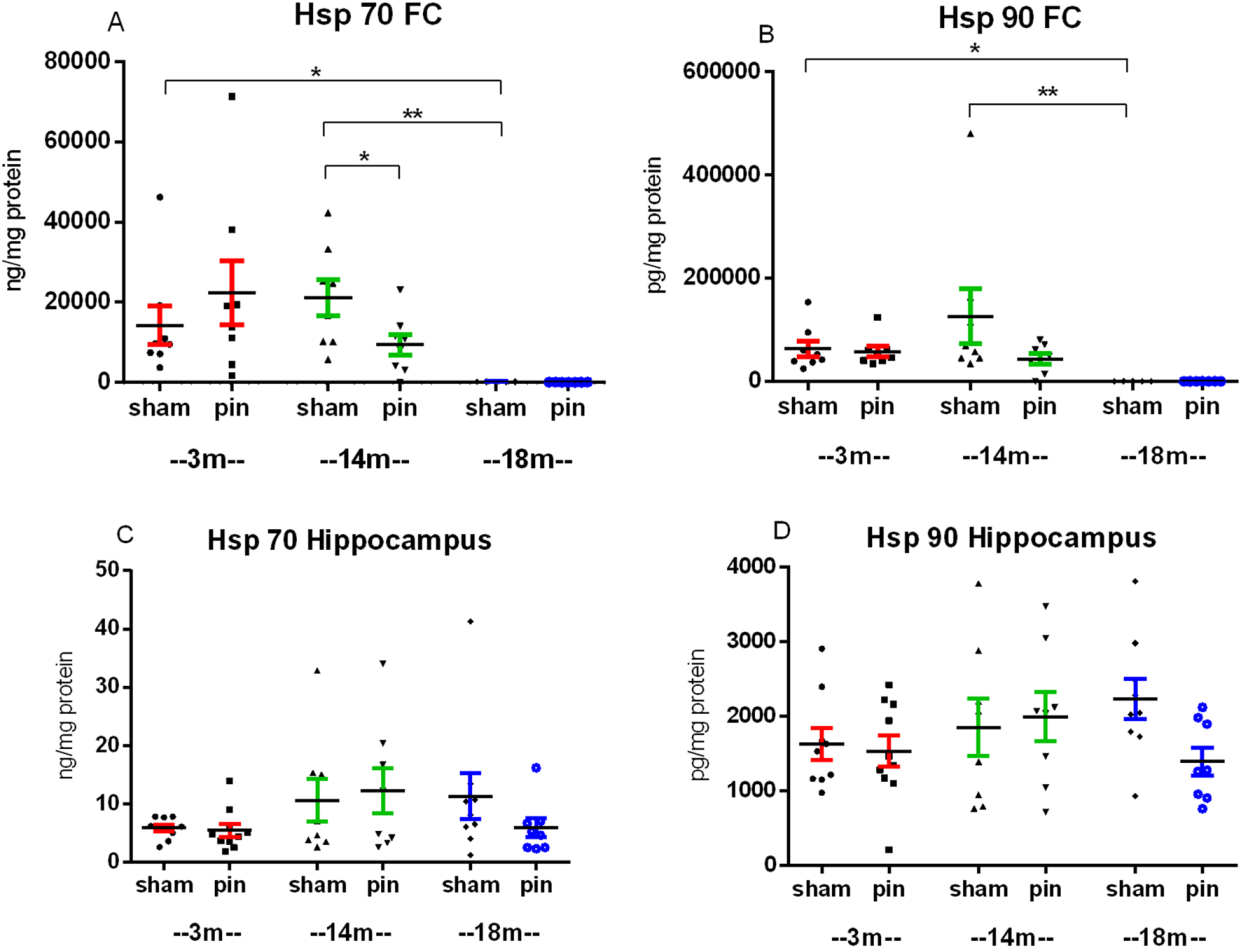
The effect of pinealectomy on the expression of the heat shock protein (Hsp) 70 **A** and Hsp90 **B** in the frontal cortex (FC) **A**, **B** and the hippocampus **C**, **D** of 3-, 14- and 18 month-old rats. Kruskal–Wallis analysis showed a main Age effect [F2,44 = 8.988, p < 0.001] and Surgery x Age [F2,44 = 4.862, p = 0.013] for the Hsp 70. A main Age effect [F2,40 = 13.552, p < 0.001] was also detected for the Hsp 90 in the FC. Data are presented as mean ± SEM. *p = 0.033, 18-month-old sham compared to 3 month-old sham rats; **p = 0.01, 18 month-old sham compared to 14 month-old sham rats; *p = 0.019, 14 month-old pin rats compared to the matched sham rats **A**. *p = 0.05, 18 month-old sham compared to 3 month-old sham rats; *p = 0.039, 18 month-old sham compared to 14 month-old sham rats **B**

The effect of the aging process and melatonin deficiency in different stages of aging on the expression of Hsp 70 and Hsp 90 in the FC and the hippocampus were analyzed to assess their role in changed emotional behavior in rats. The protective role of these proteins in helping the refolding of impaired proteins in stress conditions is well-known [20]. Age-dependent decrease in the expression of the Hsp 70 (p = 0.033, 18-month-old sham compared to 3-month-old sham rats; p = 0.01, 18-month-old sham compared to 14-month-old sham rats) and Hsp 90 (p = 0.05, 18-month-old sham compared to 3-month-old sham rats; p = 0.01, 18-month-old sham compared to 14-month-old sham rats) was detected in the FC (Fig. 8A, B). Surprisingly, pinealectomy reduced the expression of Hsp 70 in 14-month-old rats compared to the matched sham group (p = 0.019) (Fig. 8A). Similar tendency was also observed for the Hsp 90 without reaching the significance (p > 0.05) (Fig. 7B). There was no significant difference among the three studied rat generations in the expression of Hsp 70 and 90 in the hippocampus (p > 0.05) (Fig. 8C, D). Melatonin deficiency in plasma did nthese proteins in the hippocampus (p > 0.05).

## Discussion

The results of the present study demonstrated that elderly rats exhibited diminished spontaneous motor activity, increased anxiety-like behavior concomitant to a higher baseline plasma corticosterone level, and reduced expression of Hsp 70 and 90 in the FC. Pinealectomy reduced the motor activity of the young adult rats during the dark phase and blunted the diurnal activity of old rats. Further, melatonin deficiency related to pinealectomy alleviated anxiety responses to novelty without affecting the expression of Hsp 70 and 90 both in the FC and the hippocampus in young adult and old rats. The impaired activity of the HPA axis was simultaneous to the detected impulsive-like behavior in 18-month rats. The anxiety responses, the circadian rhythms of motor activity, and HPA axis activity were not affected in the middle-aged rats with pinealectomy. Still, this generation exhibited lower levels of the Hsp 70 in the FC.

Our findings for age-related changes in emotional responses in the EPM agree and extended previous reports in different rat strains [1, 2, 6]. However, other studies demonstrated contradictory results [3, 6]. Thus, Sudakov et al. [3] suggested that behavioral alterations related to emotional conditions in rats were evident as early as late adolescence (5-month-old rats). This discrepancy might not be attributed to strain differences since the authors, as mentioned above, also used Wistar rats. In the present study, we used two parameters to measure activity in the EPM (number of entries and total distance traveled), which were indicated as adequate measures of motor activity [1]. In addition, to eliminate the impact of the anxiety factor related to measurement in novelty for this parameter, we also tested motor activity for 24 h in the actimeter, suggesting that at these conditions, rats are well-habituated. The discrepancy between our data and those reported by Torras-Garcia et al. [6] can be explained by our controls being sham-operated rats. In the present study, the age-associated tendency for enhanced anxiety response was also confirmed in two additional tests for anxiety.

It is well-known that there is a close relationship between the plasma profiles of melatonin and the activity of the pineal gland because the indolamine is not stored there [14, 25]. Recently, we reported that removing the pineal gland caused abolished circadian fluctuations of melatonin levels in plasma [26], suggesting that the procedure could be considered an appropriate model of plasma melatonin deficiency. Although many other extrapineal tissues are sources of melatonin, its hormonal activity primarily involves fine-tuning endocrine and other internal “clock” signals synchronized with the external “clock” of the light–dark cycle. The tissues with the highest metabolic activity, like the brain, skin, and gut, are the most critical parts of the body where the indoleamine is also synthesized [27]. Moreover, results from animals and humans reveal that extrapineal melatonin levels can exceed the blood levels of the hormone [28–30]. Both glandular (pineal) and cellular sources of detected melatonin in the brain tissue contribute to the higher level in cerebrospinal fluid than other places [31]. Aging is accompanied by a progressive decrease in hormone production with concomitant attenuation of its protective action against oxidative stress and enhanced vulnerability to the onset of neurodegenerative diseases [25].

In the present study, we report the impact of melatonin deficiency, induced in three different generations, on anxiety-like behavior and circadian rhythm of motor activity. The role of endogenous melatonin as a hormone in anxiety behaviors was demonstrated in experimental animals and clinical conditions [32]. We found that young adult and old rats were the most vulnerable to changes in anxiety due to melatonin deficiency, showing less anxiety than the matched sham groups. In contrast, melatonin deficiency did not affect the middle-aged rats. Notably, changed emotional responses in the elderly rats with pinealectomy were not associated with altered motor activity in the novel environment. Therefore, the impaired anxiety behavior of elderly rats with pinealectomy might not be related to impulsivity as an emotional component of motor activity. Pierpaoli and Bulian [15] reported that pinealectomy harmfully affected blood, metabolic, and hormonal parameters in 3-month-old mice but not in 14-month-old rodents. Recently, we confirmed the findings of Pierpaoli and Bulian [15] that melatonin deficiency accelerates aging in young adult rats and can impair several metabolic indices with concomitant elevation of arterial blood pressure, blood glucose, triglyceride, and cholesterol [17, 18]. In the present study, the procedure of pineal gland removal in elderly rats did not confirm the hypothesis of Pierpaoli et al. [15] that pinealectomy in mice older than 14 months no longer affected the “aging program”. Although this suggestion was confirmed for the expression of Hsp 70 and 90 in the FC in the present study, surprisingly, the old rats with melatonin deficiency showed low anxiety expressed with diminished ability to react to dangerous or aversive conditions.

Moreover, unlike Pierpaoli and Bulian [15], who reported that pinealectomy accelerated aging in 3-month-old BALB/c mice with pinealectomy via a shortage of the lifespan, our results showed a lack of impact of blood melatonin deficiency on the lifespan of the youngest rats. In addition, Pierpaoli and Bulian [15] reported that middle age is a crucial period for mice, and pinealectomy caused a delay in lifespan, while melatonin deficiency in 18-month-old mice did not affect this parameter. The discrepancy between the results of Pierpaoli and Bulian [15] in mice and our findings in rats regarding the role of melatonin as a hormone on lifespan needs further clarification and is an open question. Nevertheless, our results indicate that blood melatonin deficiency might not be crucial for lifespan irrespective of the age stage of inducement, suggesting that an extra-pineal source of melatonin is enough to compensate for the lack of hormonal function.

In support of previous reports [33, 34], we demonstrated that plasma corticosterone increased with aging in rats. The discrepancy between these findings and other reports [20, 33–36] was discussed in the review of Sapolsky [33], suggesting that conflicted results might not be attributed to sex, stress, or time point of detection divergence but to methodology and procedure of blood collecting. The activity of the HPA axis is closely related to responses to stress and might be changed by aging and pathological conditions. In the present study, we report that the HPA axis-associated negative feedback control resulting from stress procedure was intact in the three generations, i.e., young adult, middle-aged, and old sham-operated rats. This finding is consistent with the report of Scaccianoce et al. [34], who demonstrated that corticosterone could suppress the release of ACTH in the pituitary in young and old rats. The adequate adrenocortical reaction after stress in aged rats was also reported by Sapolsky et al. (19,902) [34]. The increased basal corticosterone level in old rats in the present study might be due to impaired feedback control by the hypothalamus [20, 35, 37]. Age-associated melatonin deficiency contributes to high basal corticosterone levels in sham-operated 18-month-old rats compared to the matched 3-month-old rats [38]. We confirmed the result of Oxenkrug et al. [39] and demonstrated that old Wistar rats with pinealectomy had an additional elevation of corticosterone compared to the matched sham-operated rats in basal conditions while young adult and middle-aged groups were not affected. Increased plasma corticosterone from blood melatonin deficiency in elderly rats suggests a disrupted pineal control on adrenal activity.

Further, melatonin deficiency in this group led to hypoactivity of the HPA axis and inadequate response to stress stimulus. The disruption of melatonin production via removing the pineal gland, which is the primary source of this hormone, also changes the mechanism of its synthesis from immune‐competent cells responsible for inflammatory response and involved in the immune-pineal axis [40]. Interestingly, the mechanism of glucocorticoid inhibitory control by the adrenal cortex on melatonin production activated in stress conditions in intact pineal gland should be impaired in pinealectomy and possibly involved in the disturbed HPA axis activity observed in elderly rats. Recently, we reported that young adult rats with pinealectomy showed an impaired circadian rhythm of plasma corticosterone levels [26]. We can speculate that the adaptive response of immune‐competent cells in young adult and middle-aged rats with melatonin deficiency is strong enough to overcome changes in pineal-adrenal gland relationships and maintenance of the HPA axis activity. Therefore, a comparative analysis of the age-related effect of melatonin deficiency on different components of the HPA axis needs further evaluation.

The underlying molecular mechanism in the pinealectomy-induced changes in anxiety response in young adult and old rats was further evaluated by exploring the expression of Hsp 70 and Hsp 90 in homogenates from the FC and the hippocampus. The impaired mechanism of stress-induced expression of Hsp70 was suggested to be a valid biomarker of the aging process [21]. The age-related decline in the expression of Hsp 70 was reported in different rat tissues, including lymphocytes, hepatocytes, hippocampus, and FC [22, 23, 41]. Recently, we demonstrated that while a sub-chronic treatment with melatonin did not affect the expression of the Hsp 70 in basal conditions in the FC and the hippocampus in both the Wistar rats and spontaneously hypertensive rats, the hormone tended to reduce the status epilepticus-induced rise in the shock protein in the hippocampus [42]. Other authors also reported the beneficial ameliorating effect of melatonin treatment on the induction of Hsp 70 in various pathological conditions, including melatonin deficiency induced by pinealectomy [41, 43]. The age-related decline in the expression of the two Hsp (70 and 90) in the FC but not in the hippocampus reported in the present study agrees with the literature data [22, 23] and might be explained by declined hormonal function in plasma.

Conversely, the pinealectomy-induced suppression of Hsp 70 in middle-aged rats might represent an adaptive mechanism against melatonin deficiency in plasma. This suggestion aligns with Pierpaoli and Bulian (2005) hypothesis [15] that pinealectomy in 14-month-old mice might delay the aging process in some aspects. However, our recent studies in rats demonstrated that removing the pineal gland might have a harmful effect, leading to accelerated aging in middle-aged rats, suggesting a more complex role of the hormone across the lifespan. The lack of effect of pinealectomy on the Hsp 70 expression in elderly rats could also be explained by the impaired expression of these proteins in aging.

## Conclusions

In conclusion, removing the pineal gland affected emotional responses in young adult and elderly but not middle-aged rats. The impaired anxiety behavior in the oldest rats with melatonin deficiency might be related to hypoactivity of the HPA axis, resulting in hypercortisolemia. The blunted circadian rhythm of corticosterone reported in our previous study [26] might explain the disturbed emotional reactions in young adult rats with pinealectomy. The middle-aged rats with pinealectomy are a turning point where extra-pineal indolamine could compensate for the impaired hormonal function, explaining the intact emotional responses and HPA axis activity. Further research is required to estimate the precise impact of extra-pineal melatonin from CSF and the adrenal gland in conditions of blunted hormonal function of the indolamine.

## Methods

The experimental design was elaborated in accordance with the European Communities Council Directive 2010/63/ E.U. for animal experiments and approved by the Bulgarian Food Safety Agency (Research Project: # 300/N◦5888–0183).

### Animals and experimental schedule

Male Wistar rats, purchased by the vivarium of the Institute of Neurobiology, BAS), were used at three different ages: 3, 14, and 18 months old. The animals were kept in standard environmental conditions: constant 12/12 h of light/dark cycle, the average temperature of 21 ◦C, and humidity of 45%. They were in groups of 3–4 rats per cage (transparent plastic cages in different sizes depending on the age. Food and water were available ad libitum except for the testing period. The experimental design is described in Fig. 1. In brief, after surgical procedures, the rats were used in two separate protocols: Experiment#1 and Experiment#2. The rats in Experiment#1 were undisturbed after 24 h experiment in the actimeter for survival time analysis after sham/pinealectomy procedure. The rats in Experiment#2 were explored in three behavioral tests, from the least aversive to the most aversive test. After the last test, their brains were used for biochemical analysis. One cohort of animals from Experiment# 2 was utilized for the Novelty Suppression Feeding Test (NSFT), followed by decapitation. The ELISA method was employed to analyze plasma for corticosterone levels, while the frontal cortex (FC) and hippocampus were assessed for the heat shock protein (Hsp) 70 and Hsp 90. The second and third cohorts of animals from Experiment# 2 were designated for the Elevated Plus Maze (EPM) test and the Light–Dark test (LDT). After 24 h, blood was collected from the tail vein at three time points: basal (0’) on the first day, 10 min after stress (10’) on the second day, and 120 min after stress (120’). The criteria for excluding animals from testing and subsequent analysis were established to address complications arising during or after surgery that could potentially compromise the integrity of behavioral tests. Instances such as disturbances in motor function or significant weight loss were identified as critical factors warranting exclusion. These criteria were implemented to maintain the validity and reliability of the experimental data, ensuring that any observed effects were not confounded by external factors unrelated to the intended variables under investigation. The careful consideration of complications underscores the commitment to scientific rigor and the accurate interpretation of results in the study.

### Surgical procedures

Rats from each age were split into two subgroups according to the surgery procedure: sham-operated and group with pinealectomy. Anesthetized by isoflurane (2.5%), rats were fixed on a stereotaxic apparatus (Stoelting, US). They were subjected to removal of the pineal gland according to the procedure described by Hoffman and Reiter [44] and in our previous studies [19, 38].

### Measurement of spontaneous motor activity and diurnal variations

Actimeter (Infrared Actimeter, Bioseb, France, https://www.bioseb.com/en/activity-motor-control-coordination/51-infrared-actimeter.html) was applied to explore locomotion and diurnal rhythmic changes for 24 h. Each rat was tested individually in the apparatus (20 cm height, 45 cm width, and 45 cm length), and behavioral data were collected for up to 26 h. The first two hours were not included in the data analysis to gather results from well-habituated animals.

### Measurement of anxiety behavior

#### Elevated plus-maze test

The test was conducted as was shown in our previous studies [19]. In brief, each rat was placed on the central zone of the maze facing an open arm of the black wood apparatus. The following parameters were calculated during the 5 min test: total distance in the closed arms (cm), number of closed arm entries, distance, time and number of entries in open arms, distance open arms vs total arms distance, time open arms vs. total time, number entries open arms vs total arm entries. After each test, the apparatus was carefully cleaned with alcohol.

#### Light-dark test

The apparatus consisted of an open (25 cm × 50 cm × 40 cm) and a covered (dark) (25 cm × 25 cm × 40 cm) compartment, connected with a 7 cm door. The open part of the apparatus was illuminated by a bulb (80 lx) mounted over this area. At the start of the test, the rat was placed into the light compartment. The measured parameters were the total time spent in the light compartment (sec) and the latency to leaving the first time for the open compartment. The test was conducted for 5 min.

#### Novelty suppression feeding test

The hyponeophagia response to exposure of a rat to an open field (80 × 80 × 30 cm) was evaluated. The test was conducted as was described in our earlier study with little modifications [42]. Each tested rat was placed in the corner of the open field after 48 h of food deprivation. The following parameters were measured: the latency to enter the circle of a white paper inserted in the central zone, the latency to start sniffing the chow pellets placed on the paper, and the latency to begin eating for 15 min. The test was interrupted if the rats started eating the pallets or if 900 s had elapsed. As soon as the test in the OF finished, each rat was placed in its home cage, and the latency to start eating there was calculated. The hyponeophagia in the novel environment was expected to significantly increase the latency to start eating there compared to the home cage latency.

#### Corticosterone measurement

The rats from Experiment#2 were decapitated 24 h after the last behavioral test, and immediately before the decapitation, the procedure for a stress test and taking blood was done. The plasma levels of corticosterone were assessed in three different conditions, as described in our previous study [45]. In brief, the blood was collected from the tail vein before the stress procedure of forced swimming for 5 min (0’), 10 min after stress (10’), and 120 min later (120’). The blood was collected into Vacutainer^®^ with EDTA. The plasma was isolated after centrifugation at 4000 rpm for up to 10 min at 4 °C. It was kept in the refrigerator at − 70 °C until analysis. The corticosterone was measured in ng/ml by Elisa test kit (Elabscience, Lot.No FU0704H66448) following the manufacturer’s instructions.

#### Measurement of heat shock protein 70 and 90 in the frontal cortex

The FC and the two hippocampi were isolated on ice, snap-frozen into liquid nitrogen, and preserved in the fridge at − 20 °C until the biochemical assay. The tissue homogenates were prepared as described earlier [46] before analysis of the expression Hsp70 and Hsp90 by ELISA kit (Fot Ltd. Bulgaria, Lot: MBS760279; MBS7224294; Elabscience HSP70 cat No E-EL-R0479 Lot: KL05RP8D5302; Elabscience Hsp 90: Cat No: E-EL-R0480 Lot: KL03FR287799) according to the manufacture’s guidance. Bradford assay was applied for each sample to estimate the protein concentration.

### Statistical analysis

Data from survival between sham- and pin-operated rats at different ages were presented as Kaplan–Meier curves and analyzed by Log rank test. Circadian fluctuations of locomotion were analyzed by Zero amplitude test. Two-way repeated ANOVA with factors Surgery (sham/pinealectomy), and Time (24-time points) as independent factors with Bonferroni post hoc test was performed for analysis of circadian variations of motor activity. The diurnal horizontal activity was also evaluated by three-way ANOVA where the Time was replaced by Phase (light/dark) as a third factor. A two-way analysis of variance was used (factors: Age and Surgery) for anxiety tests. Data in the Hsp 70 and 90 were not normally distributed. Therefore Kruskal–Wallis on ranks followed by the Mann–Whitney U test was applied. Fisher exact test was used for analyses of data from 18 month-old rats in the NSFT. Repeated analysis of variance assessed the corticosterone level in plasma (factors: Age, Surgery and Time (0’, 10’ and 120’ after stress). The data are shown as mean ± S.E.M. SigmaStat^®^ 11.0 and GraphPad Prism 6 software were used for statistics and preparation of figures. The criterion for significant difference was accepted at p ≤ 0.05.

## Data Availability

All data generated or analysed during this study are included in this published article.

## Abbreviations

HPA: Hypothalamic-pituitary-adrenocortical
Hsps: Heat shock proteins
EPM: Elevated plus maze
LDT: Light–dark test
NSFT: Novelty-suppressed feeding test
ZT: Zeigeber
